# Nutrient-wide association study of 92 foods and nutrients and breast cancer risk

**DOI:** 10.1186/s13058-019-1244-7

**Published:** 2020-01-13

**Authors:** Alicia K. Heath, David C. Muller, Piet A. van den Brandt, Nikos Papadimitriou, Elena Critselis, Marc Gunter, Paolo Vineis, Elisabete Weiderpass, Guy Fagherazzi, Heiner Boeing, Pietro Ferrari, Anja Olsen, Anne Tjønneland, Patrick Arveux, Marie-Christine Boutron-Ruault, Francesca Romana Mancini, Tilman Kühn, Renée Turzanski-Fortner, Matthias B. Schulze, Anna Karakatsani, Paschalis Thriskos, Antonia Trichopoulou, Giovanna Masala, Paolo Contiero, Fulvio Ricceri, Salvatore Panico, Bas Bueno-de-Mesquita, Marije F. Bakker, Carla H. van Gils, Karina Standahl Olsen, Guri Skeie, Cristina Lasheras, Antonio Agudo, Miguel Rodríguez-Barranco, Maria-José Sánchez, Pilar Amiano, María-Dolores Chirlaque, Aurelio Barricarte, Isabel Drake, Ulrika Ericson, Ingegerd Johansson, Anna Winkvist, Tim Key, Heinz Freisling, Mathilde His, Inge Huybrechts, Sofia Christakoudi, Merete Ellingjord-Dale, Elio Riboli, Konstantinos K. Tsilidis, Ioanna Tzoulaki

**Affiliations:** 1 Department of Epidemiology and Biostatistics, School of Public Health, Imperial College London, St Mary’s Campus, Norfolk Place, London, W2 1PG UK; 2 Department of Epidemiology, Maastricht University Medical Centre, Maastricht, the Netherlands; 3 Department of Hygiene and Epidemiology, University of Ioannina School of Medicine, Ioannina, Greece; 4 Nutrition and Metabolism Section, International Agency for Research on Cancer, World Health Organization, Lyon, France; 5 Proteomics Facility, Center for Systems Biology, Biomedical Research Foundation of the Academy of Athens, Athens, Greece; 6 Department of Nutrition and Dietetics, School of Health Science and Education, Harokopio University, Athens, Greece; 7 International Agency for Research on Cancer, World Health Organization, Lyon, France; 8 Center of Research in Epidemiology and Population Health (CESP), Inserm U1018, Paris-South Paris-Saclay University, Gustave Roussy, Villejuif, France; 9 Department of Population Health, Luxembourg Institute of Health, Strassen, Luxembourg; 10 Department of Epidemiology, German Institute of Human Nutrition Potsdam-Rehbruecke, Nuthetal, Germany; 11 Danish Cancer Society Research Center, Copenhagen, Denmark; 12 University of Copenhagen, Copenhagen, Denmark; 13 Breast and Gynaecologic Cancer Registry of Côte d’Or, Georges-François Leclerc Cancer Centre, UNICANCER, Dijon, France; 14 Division of Cancer Epidemiology, German Cancer Research Center (DKFZ), Heidelberg, Germany; 15 Department of Molecular Epidemiology, German Institute of Human Nutrition Potsdam-Rehbruecke, Nuthetal, Germany; 16 Institute of Nutritional Sciences, University of Potsdam, Nuthetal, Germany; 17 Hellenic Health Foundation, Athens, Greece; 18 2nd Pulmonary Medicine Department, School of Medicine, National and Kapodistrian University of Athens, “ATTIKON” University Hospital, Haidari, Greece; 19 Cancer Risk Factors and Lifestyle Epidemiology Unit, Institute for Cancer Research, Prevention and Clinical Network (ISPRO), Florence, Italy; 20 Environmental Epidemiology Unit, Fondazione IRCCS Istituto Nazionale dei Tumori di Milano, Milan, Italy; 21 Department of Clinical and Biological Sciences, University of Turin, Turin, Italy; 22 Unit of Epidemiology, Regional Health Service ASL TO3, Grugliasco, Italy; 23 Dipartimento di Medicina Clinica e Chirurgia, Federico II University, Naples, Italy; 24 Department for Determinants of Chronic Diseases (DCD), National Institute for Public Health and the Environment (RIVM), Bilthoven, The Netherlands; 25 Department of Gastroenterology and Hepatology, University Medical Centre, Utrecht, The Netherlands; 26 Department of Social & Preventive Medicine, Faculty of Medicine, University of Malaya, Pantai Valley, Kuala Lumpur, Malaysia; 27 Julius Center for Health Sciences and Primary Care, University Medical Center Utrecht, Utrecht University, Utrecht, The Netherlands; 28 Department of Community Medicine, UiT The Arctic University of Norway, Tromsø, Norway; 29 Functional Biology Department, School of Medicine, University of Oviedo, Asturias, Spain; 30 Unit of Nutrition and Cancer, Cancer Epidemiology Research Program, Catalan Institute of Oncology - ICO, Group of Research on Nutrition and Cancer, Bellvitge Biomedical Research Institute – IDIBELL, L’Hospitalet of Llobregat, Barcelona, Spain; 31 Andalusian School of Public Health (EASP), Granada, Spain; 32 Instituto de Investigación Biosanitaria de Granada (ibs.GRANADA), Granada, Spain; 33 CIBER of Epidemiology and Public Health (CIBERESP), Madrid, Spain; 34 Universidad de Granada (UGR), Granada, Spain; 35 Public Health Division of Gipuzkoa, BioDonostia Research Institute, San Sebastian, Spain; 36 Department of Epidemiology, Regional Health Council, IMIB-Arrixaca, Murcia University, Murcia, Spain; 37 Navarra Public Health Institute, Pamplona, Spain; 38 Navarra Institute for Health Research (IdiSNA), Pamplona, Spain; 39 Department of Clinical Sciences in Malmö, Lund University, Malmö, Sweden; 40 Department of Odontology, Umeå University, Umeå, Sweden; 41 Department of Internal Medicine and Clinical Nutrition, Sahlgrenska Academy, University of Gothenburg, Gothenburg, Sweden; 42 Department of Public Health and Clinical Medicine, Section of Sustainable Health, Umeå University, Umeå, Sweden; 43 Cancer Epidemiology Unit, Nuffield Department of Population Health, University of Oxford, Oxford, UK; 44 MRC Centre for Transplantation, King’s College London, London, UK

**Keywords:** Breast cancer, Diet, Foods, Nutrients, Alcohol, Fibre

## Abstract

**Background:**

Several dietary factors have been reported to be associated with risk of breast cancer, but to date, unequivocal evidence only exists for alcohol consumption. We sought to systematically assess the association between intake of 92 foods and nutrients and breast cancer risk using a nutrient-wide association study.

**Methods:**

Using data from 272,098 women participating in the European Prospective Investigation into Cancer and Nutrition (EPIC) study, we assessed dietary intake of 92 foods and nutrients estimated by dietary questionnaires. Cox regression was used to quantify the association between each food/nutrient and risk of breast cancer. A false discovery rate (FDR) of 0.05 was used to select the set of foods and nutrients to be replicated in the independent Netherlands Cohort Study (NLCS).

**Results:**

Six foods and nutrients were identified as associated with risk of breast cancer in the EPIC study (10,979 cases). Higher intake of alcohol overall was associated with a higher risk of breast cancer (hazard ratio (HR) for a 1 SD increment in intake = 1.05, 95% CI 1.03–1.07), as was beer/cider intake and wine intake (HRs per 1 SD increment = 1.05, 95% CI 1.03–1.06 and 1.04, 95% CI 1.02–1.06, respectively), whereas higher intakes of fibre, apple/pear, and carbohydrates were associated with a lower risk of breast cancer (HRs per 1 SD increment = 0.96, 95% CI 0.94–0.98; 0.96, 95% CI 0.94–0.99; and 0.96, 95% CI 0.95–0.98, respectively). When evaluated in the NLCS (2368 cases), estimates for each of these foods and nutrients were similar in magnitude and direction, with the exception of beer/cider intake, which was not associated with risk in the NLCS.

**Conclusions:**

Our findings confirm a positive association of alcohol consumption and suggest an inverse association of dietary fibre and possibly fruit intake with breast cancer risk.

## Background

Dietary factors have been extensively investigated as possible risk factors for breast cancer, but overall evidence for associations is inconsistent and inconclusive [1]. Aside from alcohol intake, for which there is strong evidence of a positive association with breast cancer risk, no convincing dietary risk factors have been identified [1, 2].

Fruits and vegetables are of particular interest due to their rich content of nutrients and phytochemicals, which are thought to have anticarcinogenic effects [3]. However, epidemiological studies assessing intake of fruit and vegetables, as well as of other foods such as meat, dairy, and soy products, have yielded inconsistent results [1, 2, 4, 5]. Dietary fat intake has also been widely investigated as a possible risk factor for breast cancer because it is thought to increase endogenous oestrogen levels [6, 7]; however, there is overall limited evidence for an association [1] and results from prospective studies are conflicting [2, 6]. Based on current evidence, the 2017 World Cancer Research Fund/American Institute for Cancer Research (WCRF/AICR) Third Expert Report on diet, nutrition, physical activity, and breast cancer concluded there is suggestive but limited evidence that intake of non-starchy vegetables, carotenoid-containing foods, and diets high in calcium might be associated with a lower risk of breast cancer [1]. It is also possible that associations of foods and nutrients with breast cancer risk might differ by menopausal status [1], hormone receptor status of tumours [8], and molecular subtypes [9]. Due to inconsistencies in the existing literature, the potential role of diet in breast cancer aetiology remains unclear.

We systematically evaluated an extensive list of dietary factors in relation to breast cancer risk using a nutrient-wide association study (NWAS) approach. The NWAS takes an analogous strategy to that of genome-wide association studies (GWAS), separately estimating associations for each food and nutrient measured, and using multiple comparison adjustments to select promising associations for replication in an independent study [10]. This method has been used to investigate dietary risk associations for blood pressure [11], endometrial cancer [12], and epithelial ovarian cancer [13].

## Methods

This NWAS involved investigation of intakes of 92 foods and nutrients (for which data were available) in relation to breast cancer risk in the European Prospective Investigation into Cancer and Nutrition (EPIC) study, and calculation of the associated False Discovery Rate (FDR) to select dietary factors to evaluate in the independent replication cohort, the Netherlands Cohort Study (NLCS).

### Study populations and ascertainment of breast cancer cases

#### EPIC

The EPIC study includes 521,330 men and women aged 25 to 70 years at recruitment, which occurred between 1992 and 2000 [14]. Participants were from 23 centres in 10 European countries (Denmark, France, Germany, Greece, Italy, Norway, Spain, Sweden, the Netherlands, and the UK) and completed questionnaires on diet, lifestyle, and medical history. Informed consent was provided by all participants, and ethical approval for the study was provided by the internal review board of the International Agency for Research on Cancer and from local ethics committees in each participating country.

Women without a pre-baseline diagnosis of cancer were eligible for inclusion in these analyses; those who did not complete dietary or lifestyle questionnaires or with missing data on relevant confounders were excluded.

Incident breast cancers were identified through population-based cancer registries or active follow-up, and mortality data were obtained from cancer or mortality registries [14]. Breast cancers were classified as ICD-10 code C50. Information on oestrogen receptor (ER) and progesterone receptor (PR) status of the tumours was provided by each centre on the basis of pathology reports; this information was not available for all cases, particularly during the early years of follow-up.

#### NLCS

The NLCS includes 120,852 participants, of whom 62,573 are women, aged 55 to 69 years when recruited in 1986 from the general population in 204 municipalities in the Netherlands with computerised population registries [15]. At recruitment, participants completed a self-administered questionnaire on dietary habits, lifestyle factors, medical history, family history of cancer, and other risk factors for cancer. The NLCS was approved by the institutional review boards of the Nederlandse Organisatie voor Toegepast Natuurwetenschappelijk Onderzoek (TNO) Quality of Life research institute (Zeist, Netherlands) and Maastricht University (Maastricht, Netherlands).

For efficiency, a case-cohort approach was used for questionnaire processing and follow-up. Breast cancer cases were identified from the entire cohort, but accumulated person-years at risk in the entire cohort were estimated from a subcohort of 2589 women who were randomly sampled from the cohort immediately after recruitment. For cases and members of the subcohort, we excluded women with a prevalent cancer other than non-melanoma skin cancer at recruitment, as well as those with incomplete or inconsistent dietary data or missing confounder data.

Incident breast cancer cases were identified by record linkage to the Netherlands Cancer Registry and the Dutch National Pathology Registry.

### Dietary assessment

In the EPIC study, the diet of participants was assessed at enrolment using validated country-specific or study centre-specific dietary questionnaires or food records [14, 16, 17]. The EPIC Nutrient Database was used to calculate standardised nutrient intakes for the 10 countries [18]. All foods and nutrients in the centralised EPIC database that were available in most countries (at least eight out of ten countries; 92 dietary factors) were selected for analysis.

Information on dietary intake in the NLCS was collected at recruitment using a 150-item semi-quantitative food frequency questionnaire that estimated the average frequency and amounts of foods and beverages habitually consumed in the previous 12 months. The food frequency questionnaire has been validated and tested for reproducibility [19, 20]. Nutrient intakes were calculated by multiplying the frequency of intake by the nutrient content of specified portions based on the Dutch food composition table [21].

### Statistical analysis

Cox regression models with age as the time scale were fitted to estimate hazard ratios (HRs) and 95% confidence intervals (CIs) for risk of breast cancer as a function of each food or nutrient. In the EPIC study, age at recruitment was the entry time, and age at cancer diagnosis (except non-melanoma skin cancer), death, emigration, or last follow-up, whichever occurred first, was the exit time. In the NLCS, the total person-years at risk were estimated from the subcohort, and Prentice-weighted Cox proportional hazards models with robust standard error estimates were used to account for the case-cohort design [22]. Intakes of foods and nutrients were adjusted for energy intake using the residual method [23] and standardised (by subtracting the sample mean and dividing by the sample standard deviation (SD)) prior to modelling. HRs were estimated for a one SD increment in intake. All models were stratified by age at recruitment (5-year groups) and study centre (EPIC only) and adjusted by energy intake, history of diabetes (yes/no), educational attainment (none/primary school, technical/professional school, secondary school, longer education), smoking status (never, former, current), body mass index (BMI) (< 20, [20, 23), [23, 25), [25, 30), [30, 35), >= 35 kg/m^2^), physical activity (EPIC: Cambridge index (inactive, moderately inactive, moderately active, active) [24]; NLCS, non-occupational physical activity (≤ 30, > 30–60, > 60–90, > 90 min/day)), menopausal status at baseline (EPIC only: postmenopausal versus pre- and perimenopausal), menopausal status by BMI interaction (EPIC only), age at menopause (NLCS only), age at menarche, the interaction of parous (yes/no) and age at first pregnancy, and family history of breast cancer in mother or sister/s (NLCS only). Adjustment for factors involving menopausal status was not necessary in the replication analyses in the NLCS since all women in the NLCS were postmenopausal at baseline. We used the Benjamini-Hochberg approach to control the FDR at 0.05 [10]. The set of foods/nutrients satisfying this FDR (variables with *q* value < 0.05) within EPIC were carried forward for replication in the NLCS.

We performed the NWAS overall, as well as separately by premenopausal/postmenopausal status at baseline. Associations with breast cancer for the identified foods and nutrients in the EPIC study were also assessed by ER and PR expression in tumours for the 60% of EPIC cases and 46% of NLCS cases for whom receptor status data were available.

All analyses were performed in R version 3.6.1.

## Results

Of the 343,985 eligible women in the EPIC study without a pre-baseline diagnosis of cancer, we excluded 3343 participants who did not complete dietary or lifestyle questionnaires. A further 68,544 were excluded because they had missing values for relevant covariates, leaving 272,098 women available for analysis. In these women, 10,979 incident invasive breast cancers were identified during a median follow-up time of 15 years. After 20.3 years of follow-up, 3339 incident invasive breast cancer cases were identified in the NLCS. Women with incomplete or inconsistent dietary data (520 cases, 411 subcohort members) and those with missing data on confounders (451 cases and 326 subcohort members) were excluded, leaving 2368 invasive breast cancer cases (including 93 cases who were subcohort members) and 1608 non-case subcohort members in this analysis. Women in the NLCS subcohort were older than women in the EPIC study (mean age 61 years versus 50 years, respectively) (Tables 1 and 2). Among participants in the EPIC study, the distribution of baseline demographic characteristics did not differ substantially between breast cancer cases and non-cases (Table 1).

**Table 1:** Distribution of baseline demographic characteristics and covariates in the EPIC study

		Total		Non-case		Case	
		*n*	%	*n*	%	*n*	%
	Total	272,098	100	261,119	100	10,979	100
Age at recruitment (years)	[19.9, 40)	33,452	12	32,896	13	556	5
	[40, 45)	35,784	13	34,720	13	1064	10
	[45, 50)	52,234	19	50,102	19	2132	19
	[50, 55)	60,487	22	57,499	22	2988	27
	[55, 60)	42,506	16	40,278	15	2228	20
	[60, 65)	33,176	12	31,668	12	1508	14
	[65, 70)	10,996	4	10,580	4	416	4
	[70, 75)	2966	4	2886	1	80	1
	[75, 98.5]	497	0	490	0	7	0
Smoking status	Never	158,234	58	152,103	58	6131	56
	Former	60,085	22	57,419	22	2666	24
	Current	53,779	20	51,597	20	2182	20
Education	None/primary school	82,923	30	79,947	31	2976	27
	Technical/professional school	57,553	21	55,074	21	2479	23
	Secondary school	66,456	24	63,663	24	2793	25
	Longer education (incl. university degree)	65,166	24	62,435	24	2731	25
BMI (kg/m^2^)	[10.2, 20)	22,799	8	22,007	8	792	7
	[20, 23)	79,289	29	76,013	29	3276	30
	[23, 25)	55,581	20	53,238	20	2343	21
	[25, 30)	78,670	29	75,440	29	3230	29
	[30, 35)	26,452	10	25,443	10	1009	9
	[35, 77.9]	9307	3	8978	3	329	3
Physical activity	Inactive	60,140	22	57,932	22	2208	20
	Moderately inactive	94,409	35	90,456	35	3953	36
	Moderately active	75,196	28	72,163	28	3033	28
	Active	42,353	16	40,568	16	1785	16
Diabetes	No	265,318	98	254,607	98	10,711	98
	Yes	6780	2	6512	2	268	2
Postmenopausal	No	146,620	54	141,379	54	5241	48
	Yes	125,478	46	119,740	46	5738	52
Parous	No	42,130	15	40,579	16	1551	14
	Yes	229,968	85	220,540	84	9428	86

**Table 2:** Distribution of baseline demographic characteristics and covariates in the Netherlands Cohort Study

		Non-case		Case	
		*n*	%	*n*	%
	Total	1608	100	2368	100
Age at recruitment (years)	[55, 60)	628	39	898	38
	[60, 65)	544	34	869	37
	[65, 69]	436	27	601	25
Smoking status	Never	927	58	1306	55
	Former	339	21	552	23
	Current	342	21	510	22
Education	Primary school	516	32	701	30
	Lower vocational school	364	23	506	21
	Secondary, medium vocational school	573	36	923	39
	Higher vocational, university degree	155	10	238	10
BMI (kg/m^2^)	[14.5, 20)	81	5	77	3
	[20, 23)	377	23	511	22
	[23, 25)	457	28	633	27
	[25, 30)	555	35	931	39
	[30, 35)	118	7	181	8
	[35, 44.3]	20	1	35	1
Physical activity (non-occupational, min/day)	[0, 30]	358	22	622	26
	(30, 60]	521	32	773	33
	(60, 90]	378	24	510	22
	(90, 415]	351	22	463	20
Diabetes	Yes	57	4	74	3
Parous	Yes	292	18	479	20
Family history of breast cancer (mother/sister(s))	Yes	139	9	348	15

**Fig. 1:**
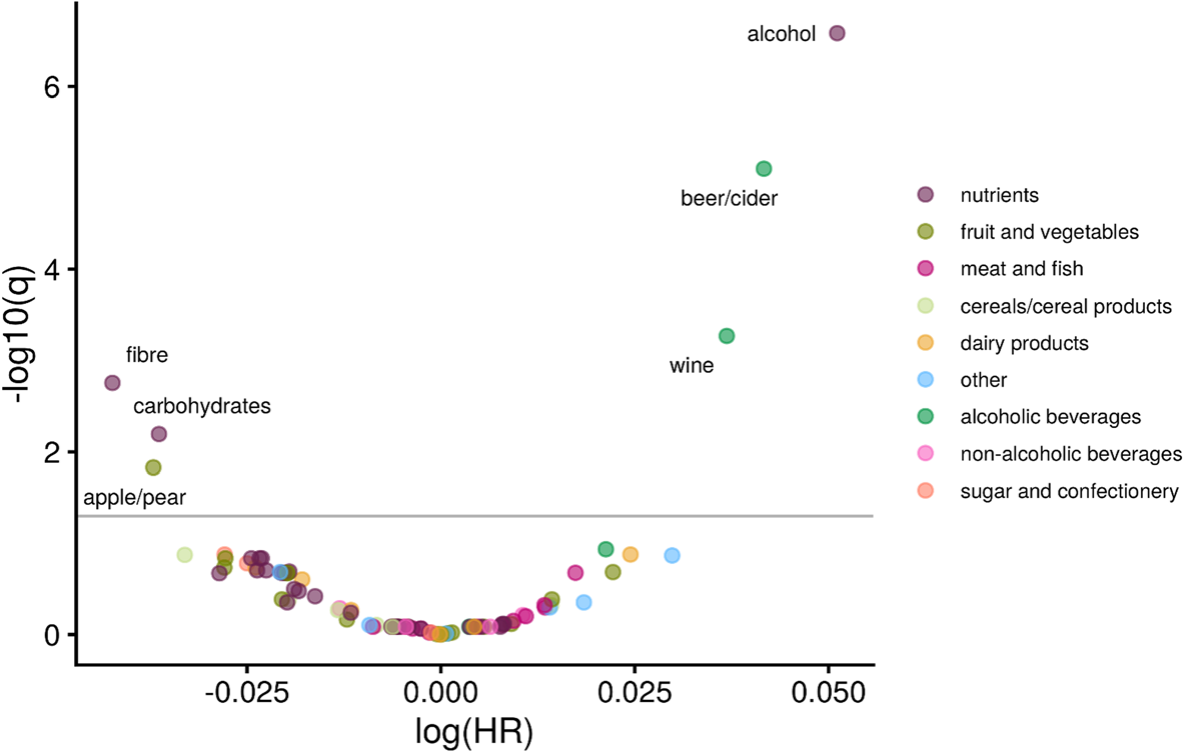
Volcano plot showing results from the nutrient-wide association study method to evaluate the association between dietary intake of 92 foods and nutrients and breast cancer risk in the EPIC study. The *y*-axis shows the negative log_10_ transformation of the estimated *q* values from the multivariable adjusted Cox proportional hazards regression coefficients, and the *x*-axis is the estimated log hazard ratio for a one standard deviation increment in intake in relation to risk of breast cancer. The *q* values represent the adjusted *p* values using the false discovery rate method, and the horizontal line indicates the false discovery rate threshold of 0.05. Each dietary factor was analysed one at a time, and ordered left to right according to the lowest to highest HR. Models were stratified by age at recruitment and study centre and adjusted for energy intake, history of diabetes, educational attainment, smoking status, BMI, physical activity, menopausal status at baseline, menopausal status by BMI interaction, age at menarche, and the interaction of parous (yes/no) and age at first pregnancy. The six dietary factors that were selected for confirmation in the NLCS are labelled

The mean (SD) intakes of the 92 foods and nutrients that were evaluated in the EPIC study are presented in Additional file 1. Of these foods and nutrients, six were associated with risk of breast cancer when controlling the FDR at 0.05 (Fig. 1). Higher intakes of alcohol, beer/cider, and wine were associated with a higher risk of breast cancer (HRs for a 1 SD increment in intake = 1.05, 95% CI 1.03–1.07, 1.05, 95% CI 1.03–1.06, and 1.04, 95% CI 1.02–1.06, respectively), whereas higher fibre, apple/pear, and carbohydrate intakes were associated with a lower risk of breast cancer (HRs per 1 SD increment in intake = 0.96, 95% CI 0.94–0.98; 0.96, 95% CI 0.94–0.99; and 0.96, 95% CI 0.95–0.98, respectively). Model estimates for the 92 dietary factors are provided in Additional file 2. In a model with mutual adjustment for intakes of fibre, apple/pear, and carbohydrates, the associations were slightly weaker: HRs per 1 SD increment in intake were 0.98, 95% CI 0.95–1.00 for fibre; 0.98, 95% CI 0.96–1.00 for apple/pear; and 0.98, 95% CI 0.96–1.00 for carbohydrate).

**Fig. 2:**
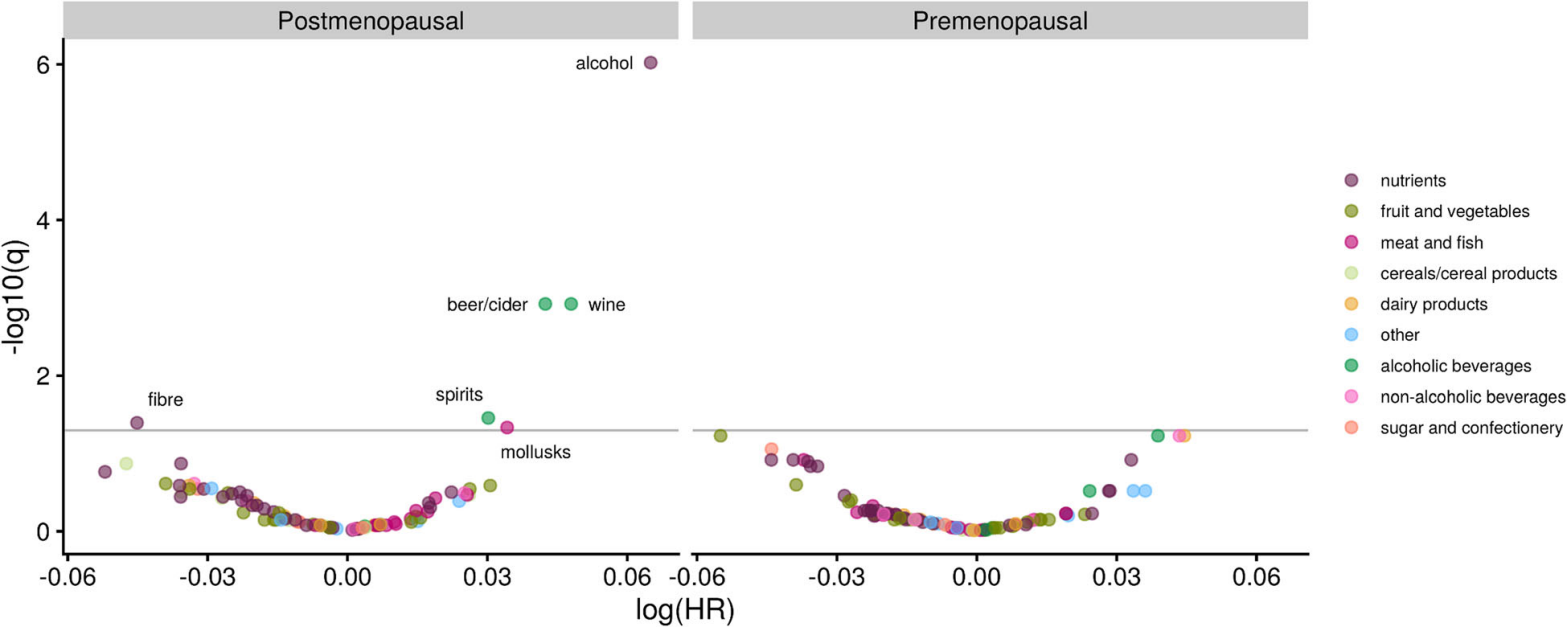
Volcano plot of estimates and *q* values for 92 foods and nutrients in relation to breast cancer risk, for the nutrient-wide association study run separately by menopausal status at baseline in the EPIC study. The *y*-axis is the negative log_10_ transformation of the estimated *q* value, and the *x* axis is the estimated log hazard ratio for a one standard deviation increment in intake. The horizontal line indicates the false discovery rate threshold of 0.05. Estimates are from Cox regression models stratified by age at recruitment and study centre and adjusted for energy intake, history of diabetes, educational attainment, smoking status, BMI, physical activity, age at menarche, and the interaction of parous (yes/no) and age at first pregnancy. Variables that met the FDR threshold are labelled

**Fig. 3:**
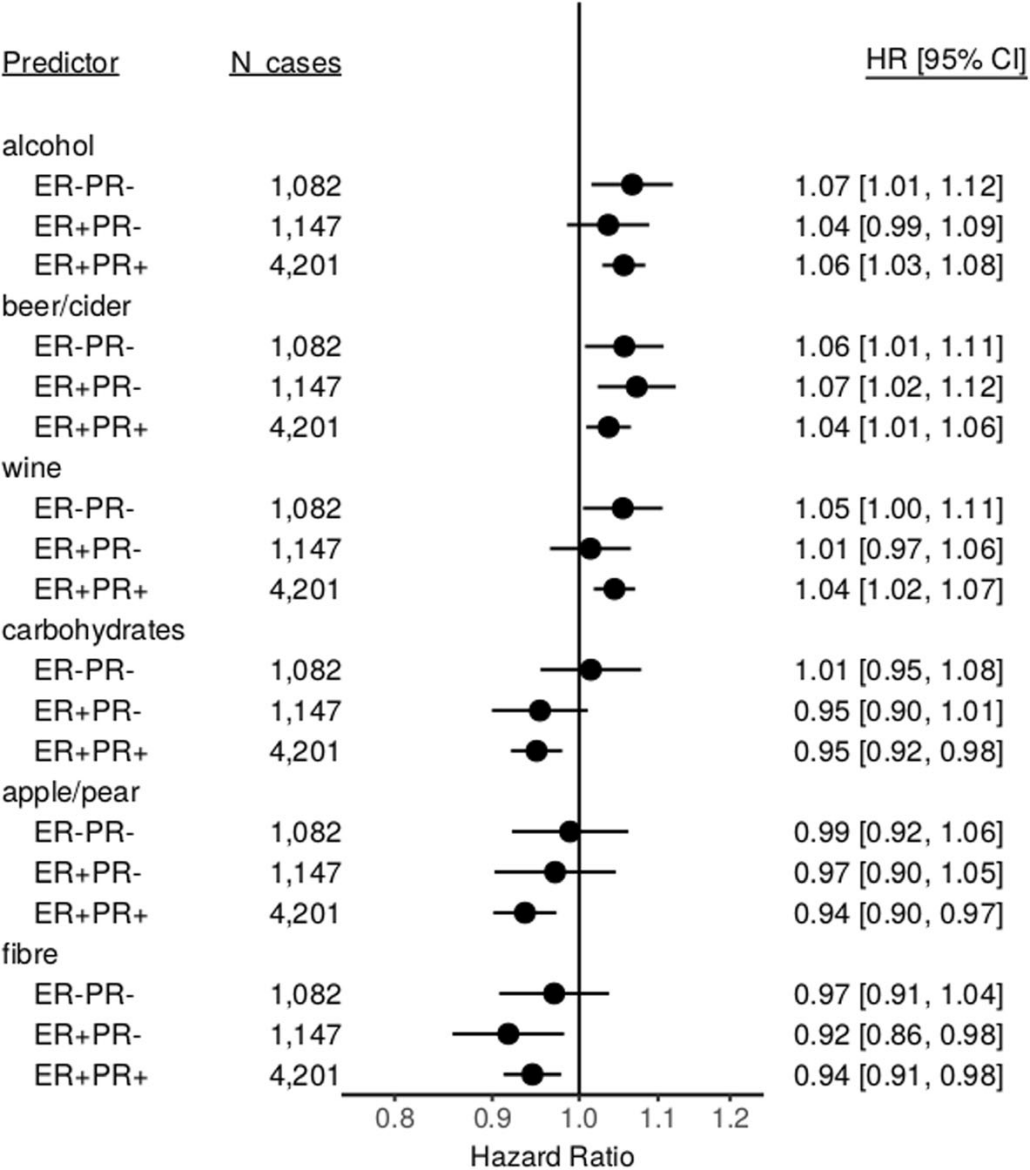
Estimated hazard ratios and 95% confidence intervals for six foods and nutrients in relation to breast cancer risk by hormone receptor status in the EPIC study. Estimates are from Cox regression models stratified by age at recruitment and study centre and adjusted for energy intake, history of diabetes, educational attainment, smoking status, BMI, physical activity, menopausal status at baseline, menopausal status by BMI interaction, age at menarche, and the interaction of parous (yes/no) and age at first pregnancy. There was an insufficient number of ER−/PR+ cases to allow separate estimation

In separate analyses by menopausal status, alcohol, beer/cider, and wine intakes were associated with a greater risk, and fibre intake was associated with a lower risk of breast cancer among postmenopausal women (*N* = 5738 cases) but not among premenopausal women (*N* = 5241 cases) (Fig. 2). We also found intakes of spirits and molluscs (both associated with higher risk) met the FDR threshold among postmenopausal women. None of the other foods or nutrients met the FDR threshold among either postmenopausal or premenopausal women. There were no substantial differences in the magnitude of the associations of alcohol, beer/cider, wine, fibre, apple/pear, and carbohydrates with breast cancer risk by hormone receptor status of tumours, with the possible exception of apple/pear and carbohydrate intake, which may have no association with risk of ER/PR negative tumours (Fig. 3).

**Fig. 4:**
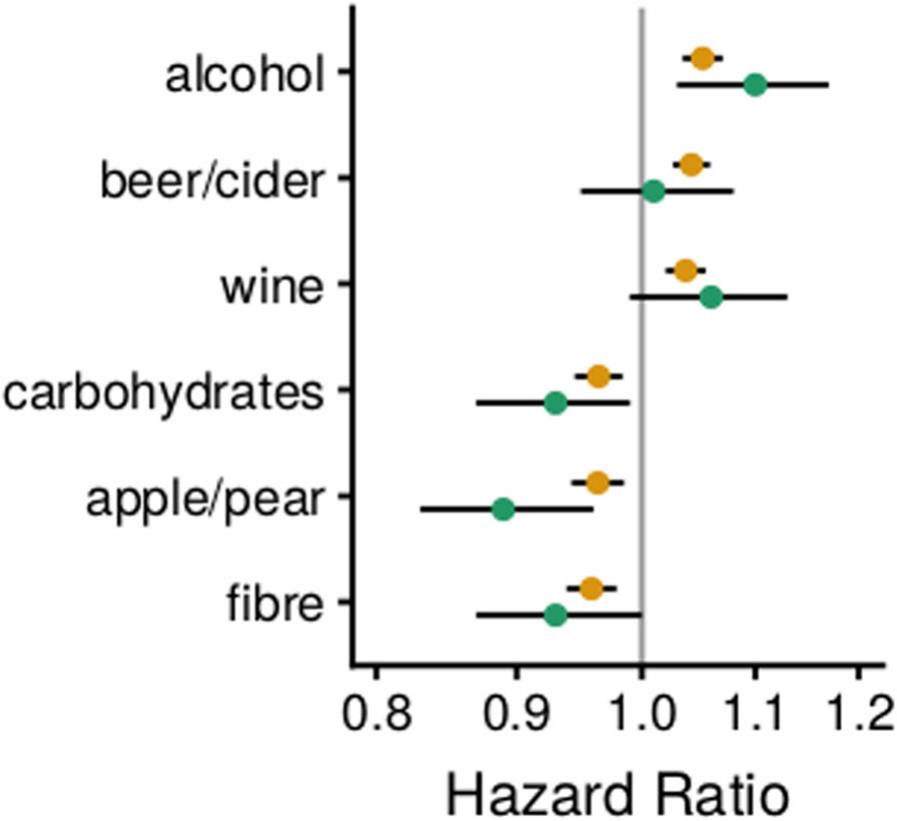
Estimated hazard ratios and 95% confidence intervals for six foods and nutrients in relation to risk of breast cancer from the EPIC analysis (yellow) and the replication in the NLCS (green). Estimates are from Cox regression models stratified by age at recruitment and study centre (EPIC only) and adjusted for energy intake, history of diabetes, educational attainment, smoking status, BMI, physical activity, menopausal status at baseline (EPIC only), menopausal status by BMI interaction (EPIC only), age at menopause (NLCS only), age at menarche, the interaction of parous (yes/no) and age at first pregnancy, and family history of breast cancer in mother or sister/s (NLCS only)

In the NLCS, we evaluated the six dietary factors that were identified in the EPIC study overall. The magnitude and direction of the association observed in the NLCS was similar to that in EPIC for each of the factors, with the exception of beer/cider intake, which was not associated with risk of breast cancer in the NLCS (Fig. 4). The NLCS results did not vary appreciably by ER/PR status (data not shown).

## Discussion

We used the NWAS approach to evaluate dietary intakes of 92 foods and nutrients in the EPIC study and identified three dietary factors (alcohol, beer/cider, wine) for which higher consumption was associated with higher risk, and three dietary factors (fibre, apple/pear, carbohydrates) for which higher intake was associated with lower risk of breast cancer (FDR < 0.05). The positive association of alcohol, and inverse associations of fibre, apple/pear, and carbohydrate intake with breast cancer risk were confirmed in the NLCS.

In the EPIC study, associations of the identified foods and nutrients with breast cancer risk did not differ substantially by hormone receptor status, but intakes of apple/pear and carbohydrates appeared to have no association with risk of ER/PR-negative tumours. Our analyses stratified by menopausal status showed that results for postmenopausal women alone were very similar to the overall results. No foods or nutrients met the FDR threshold when analysis was restricted to premenopausal women. More research is needed to verify the lack of findings for premenopausal breast cancer and to evaluate underlying mechanisms.

Advantages of this study include its large size and long duration of follow-up, and the NWAS approach which involved a comprehensive assessment of foods and nutrients whilst accounting for multiplicity of tests, and replication of findings in an external cohort. Possible explanations for the lack of consistent associations between dietary factors and breast cancer in epidemiological studies include measurement error arising from the dietary assessment method, and inadequate dietary variation or narrow range of intakes in individual studies. Moreover, associations for dietary factors, if they do exist, may be of a small magnitude for which many studies have had inadequate statistical power to detect. A strength of the EPIC study is the variation in diet. The wide range of dietary intakes of foods and nutrients in this heterogeneous population, a key aspect of the study design [14, 25], and large number of cases provided sufficient statistical power to detect weak to moderate associations. The primary limitation of our study is that it relied on a single assessment of dietary intake during adulthood. In addition, there was no mutual adjustment for other dietary factors (except for the model mutually adjusted for fibre, apple/pear, and carbohydrate intakes), and intercorrelations and overall dietary patterns were not accounted for in these analyses. This was merely an exploratory investigation to identify which dietary factors are associated with breast cancer, so that these factors can subsequently be evaluated in-depth in focused analyses with adjustment for other dietary confounders and to evaluate interrelationships between these foods and nutrients in greater detail***.*** Further, whilst the analogy to GWAS is somewhat appropriate, especially in terms of the approach to statistical analysis, it is far from perfect. The variants typed on a genome-wide array are typically not chosen because of any hypothesised association, but rather to provide adequate coverage of genetic variation in the whole genome. On the other hand, the 92 foods and nutrients in our study were assessed and derived and made available in the EPIC database because of prior plausibility of their association with disease outcomes. Further, the food and nutrient intakes are not independent. Thus, the NWAS approach is more closely aligned to a systematic analysis of candidate genes than it is to the hypothesis-agnostic approach of GWAS.

This study reaffirms the well-established positive association between alcohol intake and breast cancer risk [1, 26–28] and, in particular, adds to the strong, convincing evidence that alcohol consumption increases the risk of postmenopausal breast cancer [1, 28, 29]. In the EPIC study there was a positive association between alcohol intake and ER−/PR− and ER+/PR+ breast cancer. The association for ER+/PR− breast cancer was of similar magnitude and in the same direction. In a pooled analysis of 20 prospective cohort studies (as part of the Pooling Project of Prospective Studies of Diet and Cancer), alcohol consumption was positively associated with all three of these subtypes [28]. The positive association between beer/cider intake and breast cancer risk in the EPIC study was not replicated in the NLCS, perhaps due to the low beer consumption of this elderly female Dutch cohort [30]. Overall, there is compelling evidence that alcohol intake increases the risk of breast cancer.

In this NWAS, inverse associations between dietary fibre and carbohydrate intake and breast cancer risk were identified and confirmed in the independent NLCS cohort. The 2017 WCRF/AICR Continuous Update Project report concluded that there is only limited evidence, for which no conclusions can be drawn, for associations of dietary fibre and carbohydrate intake with risk of breast cancer [1]. For fibre intake, findings from epidemiological studies have thus far been inconsistent, but recent meta-analyses have found inverse associations of small magnitude, that did not differ by menopausal status or geographical region [31–33].

The inverse association between total dietary fibre intake and breast cancer risk in the EPIC study has been reported previously [34]; however when considering sources of fibre, this association was largely driven by an inverse association with fibre from vegetables, and possibly fruit, but not fibre from cereals or other dietary sources [34]. Dietary fibre intake was inversely associated with breast cancer risk in the Million Women Study in the UK (29,005 breast cancer cases in 691,571 postmenopausal women; relative risk (RR) per 5 g/day higher intake = 0.91, 99% CI 0.87–0.96); the association was evident for intake of fibre from fruit but not from vegetables or cereals [29]. In a meta-analysis of 16 prospective studies including 26,523 breast cancer cases in 999,271 participants, higher total dietary fibre intake was associated with a slightly lower risk of breast cancer (summary RR for high versus low intake = 0.93, 95% CI 0.89–0.98), but when considering source of fibre, the inverse association was apparent for soluble fibre but not for insoluble, vegetable, fruit, or cereal fibre [31]. It has not been established whether fibre from specific food sources is more beneficial than other sources, although it is possible that fibre intake in general is protective, irrespective of the specific food source. Few studies have investigated the association of dietary fibre with breast cancer risk by hormone receptor status, and results have been inconsistent [29, 31, 35, 36]. Similar to a previous analysis in the EPIC study [34], we found little variation in the association of dietary fibre intake with breast cancer risk by hormone receptor status.

The inverse association for apple/pear intake found in the current analysis could be reflecting fibre intake but might not be solely due to the fibre content of these fruits. Indeed, the association was slightly weaker but persisted after adjustment for fibre and carbohydrate intake. We speculate that it is possible that apple/pear intake is indicative of fruit intake in general since these are commonly consumed fruits in Western populations, and thus, their intake may be well captured in dietary questionnaires. In the EPIC study, apples and pears made the greatest contribution to total fruit intake [37]. In a meta-analysis of 10 prospective cohort studies, higher fruit intake was associated with a slightly lower risk of breast cancer (summary RR for highest versus lowest intake = 0.92, 95% CI 0.86–0.98) [38]. Fruit intake was also inversely associated with breast cancer risk in the Million Women Study (RR per 100 g/day higher intake = 0.94, 99% CI 0.92–0.97) [29]. Despite this, we found no strong evidence that total fruit intake was associated with breast cancer risk, which is consistent with previous analyses of EPIC data [37]. The converse scenario is therefore possible: that intake of apples and pears themselves may be associated with risk of breast cancer and that the observed associations for total fruit intake in some studies could be reflecting apple/pear intake. In a pooled analysis of 20 prospective cohort studies, total fruit intake was not associated with breast cancer risk (pooled RR for highest versus lowest quintile = 0.99, 95% CI 0.95–1.03), but intake of apples/pears was inversely associated with risk of ER− breast cancer (pooled RR per serving (138 g)/day = 0.92, 95% CI 0.85–0.99) [8]. In our study, the inverse association of apple/pear intake was most apparent for ER+/PR+ breast cancer. The potential mechanism by which specifically apple/pear intake might be associated with breast cancer risk is unclear.

The inverse association of carbohydrate intake with breast cancer risk in this NWAS could be, at least in part, due to total carbohydrate intake capturing fibre and fruit consumption. Notably, the magnitude of the association for carbohydrates was identical to that for fibre and apple/pear intake. In addition, in the EPIC study, fruit was the second biggest food group source of carbohydrates (contributing 13%) [39]. Nevertheless, after adjusting for intakes of apple/pear and fibre, the association for carbohydrate intake was weaker but did not disappear. Total carbohydrates also comprise other foods including bread (which contributed the highest proportion of carbohydrates in EPIC [39, 40]), grains, cereals, dairy products, legumes, and vegetables, but none of these dietary factors were associated with risk of breast cancer in our study. Total carbohydrate intake is also reflective of overall dietary pattern, which might be more pertinent than individual foods/nutrients for breast cancer risk.

Vegetables have garnered interest due to their rich phytochemical content and have been widely investigated for possible associations with breast cancer. In our study, no individual vegetables nor vegetable groups were associated with risk of breast cancer. Consistent with our results, a meta-analysis of 10 prospective studies [38], and a pooled analysis of 20 cohort studies [8], did not find any association between total vegetable intake and overall breast cancer risk, and likewise, no clear association was found in the Million Women Study [29]. Several studies, including a previous analysis of EPIC data [37], have found an inverse association of total vegetable intake with breast cancer risk, which was most apparent for ER−/PR− tumours [9]. The 2017 WCRF/AICR report concluded that there is suggestive but limited evidence that intake of non-starchy vegetables might decrease the risk of ER− breast cancer [1]. The report also stated there is limited suggestive evidence that consumption of foods (i.e. some fruits and vegetables) containing carotenoids decreases the risk of breast cancer [1]. Given the inconsistencies in the literature regarding the role of fruit and vegetable intake in prevention of breast cancer, no firm conclusions can be drawn at present. Nevertheless, fruits and vegetables contain numerous nutrients, as well as fibre, which might collectively protect against cancer, rather than conferring a protective effect in isolation [3].

Previous analyses in the EPIC study have found a weak association between saturated fat intake and breast cancer risk [41, 42], whereas no associations for total dietary fat intake or subtypes of fat intake were found in the present analysis. The lack of associations using this systematic NWAS approach, and in several other cohort studies [2, 29], suggests that dietary fat is unlikely to play an important role in breast cancer aetiology. However, a limitation of observational studies is that dietary questionnaires are limited in assessing eating out behaviours, and high fat processed foods consumed out of home might not be fully captured.

The fact that few foods and nutrients were found to be associated with breast cancer risk in this study, and other studies [1, 6, 29], could support suggestions that diet in middle-age, or relatively recent diet, might not play an important role in the development of breast cancer [26]. It remains unclear whether diet throughout the life course or potential windows of susceptibility, for example during childhood and adolescence, is associated with breast cancer risk. However, it is worth noting that consistent with the dietary factors identified in this study, fibre intake and apple intake during adolescence and early adulthood were inversely associated with breast cancer risk in the Nurses’ Health Study II [43, 44].

The associations identified in this study are supported by biologically plausible mechanisms. In particular, it is thought that dietary fibre intake may exert a beneficial effect for prevention of breast cancer by decreasing circulating oestrogen levels via inhibition of intestinal reabsorption of oestrogens excreted in bile and concomitant increased faecal excretion of oestrogens [45–48]. Alcohol has been shown to increase circulating concentrations of sex steroids, particularly oestrogens [49–51], and thus, the effect of alcohol on breast cancer risk is also thought to be at least partially mediated by an effect on endogenous sex hormone levels [27, 50]. Nevertheless, the mechanisms by which alcohol consumption increases breast cancer risk are poorly understood, and other potential pathways include the effect of alcohol on folate absorption [2], acetaldehyde production, oxidative stress, and epigenetic alterations [27].

## Conclusions

This study confirms the well-established increased risk of breast cancer associated with alcohol consumption and suggests that higher intake of dietary fibre and possibly fruit might be associated with reduced breast cancer risk.

## Supplementary information

**Additional file 1:** Means and standard deviations. Mean intake and standard deviation of the 92 foods and nutrients that were evaluated in the EPIC study.

**Additional file 2:** Model estimates. Estimates from the models for the 92 dietary factors that were evaluated in the EPIC study.

## Data Availability

For information on how to submit an application for gaining access to EPIC data and/or biospecimens, please follow the instructions at http://epic.iarc.fr/access/index.php

## Abbreviations

BMI: Body Mass Index
CI: Confidence interval
EPIC: European Prospective Investigation into Cancer and Nutrition
ER: Oestrogen receptor
FDR: False discovery rate
GWAS: Genome-wide association study
HR: Hazard ratio
NLCS: Netherlands Cohort Study
NWAS: Nutrient-wide association study
PR: Progesterone receptor
RR: Relative risk
SD: Standard deviation
WCRF/AICR: World Cancer Research Fund/American Institute for Cancer Research
